# Clinical and economic burden of comorbid coronary artery disease in patients with acute exacerbation of chronic obstructive pulmonary disease: sex differences in a nationwide cohort study

**DOI:** 10.1186/s12931-022-01945-7

**Published:** 2022-02-12

**Authors:** Yanan Cui, Zijie Zhan, Yiming Ma, Ke Huang, Chen Liang, Xihua Mao, Yaowen Zhang, Xiaoxia Ren, Jieping Lei, Yan Chen, Ting Yang, Chen Wang

**Affiliations:** 1 Department of Respiratory and Critical Care Medicine, The Second Xiangya Hospital of Central South University, Changsha, Hunan China; 2 Department of Pulmonary and Critical Care Medicine, China-Japan Friendship Hospital, Beijing, China; 3 National Clinical Research Center for Respiratory Diseases, Beijing, China; 4 Institute of Respiratory Medicine, Chinese Academy of Medical Science, Beijing, China; 5 Chinese Alliance for Respiratory Diseases in Primary Care, Beijing, China; 6 Chinese Academy of Medical Sciences and Peking Union Medical College, Beijing, China

**Keywords:** AECOPD, Coronary artery disease, Sex, Clinical outcomes

## Abstract

**Background:**

Coronary artery disease (CAD) is a common comorbidity of chronic obstructive pulmonary disease (COPD). However, data related to the impact of CAD on outcomes of acute exacerbation of COPD (AECOPD) are limited and whether the relationship depends on sex remains unknown. Our aim was to determine the impact of comorbid CAD on clinical outcomes among men and women with AECOPD.

**Methods:**

We used data from the acute exacerbation of chronic obstructive pulmonary disease inpatient registry (ACURE) study, which is a nationwide observational real-world study conducted between September 2017 and February 2020 at 163 centers in patients admitted with AECOPD as their primary diagnosis. Patients were stratified according to the presence or absence of CAD in men and women. The primary outcomes were the length of hospital stay and economic burden during hospitalization.

**Results:**

Among 3906 patients included in our study, the prevalence of CAD was 17.0%, and it was higher in women than in men (19.5% vs. 16.3%; P = 0.034). Age and other cardiovascular diseases were common factors associated with comorbid CAD in men and women, while body-mass index, cerebrovascular disease, and diabetes were determinants in men and pre-admission use of long-acting beta-adrenoceptor agonist and home oxygen therapy were protective factors in women. Only in men, patients with CAD had a longer length of hospital stay (median 10.0 vs. 9.0 days, P < 0.001), higher total cost during hospitalization (median $1502.2 vs. $1373.4, P < 0.001), and more severe COPD symptoms at day 30 compared to those without CAD. No significant difference was found in women. Comorbid CAD showed no relationship with 30-day readmission or death regardless of sex. In our real-world study, mortality/readmission risk within 30 days increased in patients with previous frequent hospitalizations and poorer pulmonary function.

**Conclusions:**

In hospitalized AECOPD patients, comorbid CAD was significantly associated with poorer short-term outcomes in men. Clinicians should have heightened attention for men with comorbid CAD to achieve an optimal management of AECOPD patients.

**Supplementary Information:**

The online version contains supplementary material available at 10.1186/s12931-022-01945-7.

## Background

Chronic obstructive pulmonary disease (COPD) is a major cause of morbidity and mortality worldwide that induces a considerable economic and social burden [1]. Comorbidities are common at any severity of COPD that may have a significant impact on disease process [1]. Among hospitalized population with an acute exacerbation of COPD (AECOPD) and severe airflow limitation, severe coronary artery disease (CAD) is present in about one-third of patients [2]. Moreover, patients with reduced lung function are at higher risk of developing CAD [3]. Early identification and proper management of these patients might improve the prognosis and reduce the risk of death.

Patients with COPD and CAD have specific clinical characteristics including more frequent respiratory symptoms, worse health status, and higher medication expenditure [4–6]. However, researches about the impact of comorbid CAD on clinical outcomes in AECOPD patients are limited. A single center historical cohort study including 507 separate hospital admissions for AECOPD reported that comorbid CAD was associated with longer length of stay, greater risk of intensive care unit (ICU) admission, and death [7]. This study has a relatively small sample size and did not investigate the impact of CAD on symptoms, economic burden of hospitalization, and future exacerbations or mortality among AECOPD patients.

Many studies have shown differences between men and women regarding the prevalence and clinical characteristics of comorbidities such as cardiovascular disease (CVD), bronchiectasis, and metabolic syndrome in COPD [8–10]. A multicenter study of patients hospitalized for COPD exacerbations showed that women had a lower prevalence of CAD but presented more chronic heart failure [11]. Instead, a 10-year study in subjects hospitalized due to COPD in Beijing reported that the prevalence of CAD decreased with years in men but increased in women, and reached to be similar between men and women at the end of the study [12]. The burden of CAD in the current Chinese AECOPD population remains unclear. Furthermore, scarce data exist in analyzing the sex-related differences in AECOPD outcomes according to the presence or absence of CAD.

This multicenter real-world study aimed to investigate the predictors of comorbid CAD in AECOPD patients according to sex and assess the effect of CAD on the length of hospital stay, healthcare costs, and change of symptom score during hospitalization in men and women among patients admitted for AECOPD. We additionally aimed to study whether the contribution of comorbid CAD to symptoms and readmissions or death within 30 days after discharge differed by sex and explore the factors associated with readmissions or death after 30 days.

## Methods

### Study design and patients

This study analyzed data from the acute exacerbation of chronic obstructive pulmonary disease inpatient registry (ACURE) study. The ACURE study is an ongoing, nationwide multicenter, observational patient registry designed to investigate the clinical characteristics, treatments, and prognoses of Chinese patients admitted for AECOPD in a real-world setting. It started from September 2017 and planned to recruit 7600 hospitalized COPD patients due to exacerbation with a 3-year follow-up. Details of the ACURE study design have been previously described [13]. The study was approved by the ethics committee of China-Japan Friendship Hospital (No. 2015-88) and informed consent was obtained from all involved participants. This study was conducted in accordance with the Declaration of Helsinki.

**Fig. 1:**
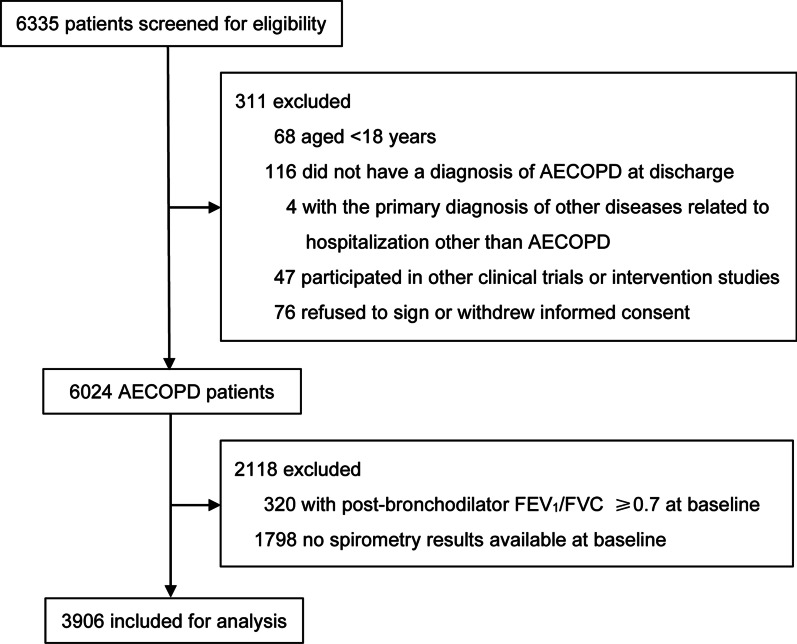
Flowchart of patient enrollment. *AECOPD* acute exacerbations of chronic obstructive pulmonary disease, *FEV*_*1*_ forced expiratory volume in 1 s, *FVC* forced vital capacity

Data on February 25th, 2020 (Phase I) in the ACURE study from 163 centers were reviewed in our study. The eligibility criteria for AECOPD patients were: (1) ≥ 18 years old; (2) inpatients with a primary diagnosis of AECOPD; (3) the presence of post-bronchodilator forced expiratory volume in 1 s (FEV_1_)/forced vital capacity (FVC) ratio < 0.70 at baseline. Patients were excluded if they participated in other clinical trials or withdrew the informed consent. The flow chart of patient enrollment is shown in Fig. 1. We grouped the study population according to sex and the status of CAD.

### Measurements

At admission, we extracted the baseline characteristics including age, sex, body-mass index (BMI), smoking status, the number of hospital or emergency admissions in the previous year, symptoms, modified Medical Research Council (mMRC) dyspnea grade, COPD Assessment Test (CAT) score, pulmonary function, pre-admission medications, and pre-admission non-drug therapy. Data on heart rate, respiratory rate, blood tests, drug treatment, intensive care unit (ICU) admission, length of hospital stay, total cost during hospitalization, and CAT score at discharge were all collected. Total costs were shown in US dollars using the average exchange rate in 2019 (one US dollar was equivalent to 6.90 yuan). We recorded the mMRC dyspnea grade, CAT score, and St George’s Respiratory Questionnaire (SGRQ) score at day 30 after discharge and readmissions or death within 30 days following discharge.

Comorbidities confirmed based on patient history, symptoms, and relevant examinations or former diagnoses according to the historical clinical records were also reviewed, including respiratory disease (asthma, bronchiectasis, lung cancer, pulmonary artery hypertension, sleep apnea syndrome, and pneumonia), CVD (CAD, hypertension, acute or chronic heart failure, and arrhythmia), digestive disease (gastroesophageal reflux disease, peptic ulcer, and chronic gastritis), cerebrovascular disease, endocrine and metabolic disease (diabetes and osteoporosis), and other malignant tumors. CAD was defined as stable angina pectoris, unstable angina pectoris, and myocardial infarction in accordance with relevant diagnostic criteria [14–16] or former diagnosis of CAD.

### Outcomes

The primary outcomes were the length of hospital stay and cost during hospitalization. The secondary outcomes were the change of CAT score at discharge compared to admission, mMRC dyspnea grade at day 30, CAT score at day 30, SGRQ score at day 30, all-cause readmission or death within 30 days, and AECOPD readmission within 30 days.

### Statistical analyses

Medians (interquartile range, IQR) or means (standard deviation, SD) were used to describe continuous data, and frequencies (percentage) were calculated for categorical data. The continuous variables were compared using the Mann–Whitney U test and categorical variables were compared using the chi-squared test or Fisher’s exact test. Multivariate logistic regression analyses (baseline variables were considered if they showed a *P*-value < 0.10 in univariate analyses) were performed to identify independent risk factors associated with comorbid CAD in men and women.

Cox proportional hazard regression model was used to determine variables associated with all-cause readmission or death within 30 days or AECOPD readmission within 30 days. We included the following clinically significant variables when studying potential in the univariate analyses: sex, status of CAD, age, BMI, smoking status, hospital admissions in the previous year, mMRC dyspnea grade and CAT score at admission, post-bronchodilator FEV_1_% predicted, comorbidities, blood gas, neutrophil-to-lymphocyte ratio (NLR), eosinophil count, N-terminal pro-B-type natriuretic peptide (NT-proBNP), C-reactive protein (CRP), procalcitonin (PCT), and drug treatment during hospitalization. Each variable was initially tested individually before we added those variables having a univariate association (*P* < 0.10) to the multivariate model. All analyses were carried out using the software IBM-SPSS statistics 25. Unless stated otherwise, a two-sided *P*-value < 0.05 was considered statistically significant.

## Results

### Baseline characteristics

Of the 3906 subjects included in the study, 664 (17.0%) had comorbid CAD. Baseline characteristics of the study patients are shown in Table 1. AECOPD patients combined with CAD were older, more likely to be ex-smokers, and had a higher BMI. Moreover, comorbid CAD was associated with increased cough, a higher CAT score, and more cardiovascular, cerebrovascular, and diabetic comorbidities. There was no difference in the post-bronchodilator FEV_1_% predicted, although the median FEV_1_/FVC ratio seemed to be higher in patients with CAD.

**Table 1:** Baseline characteristics of the study patients according to status of coronary artery disease and sex

Variables	CAD (n = 664)	No CAD (n = 3242)	*P*-value	Men			Women		
				CAD (n = 500)	No CAD (n = 2564)	*P*-value	CAD (n = 164)	No CAD (n = 678)	*P*-value
Age (years)	73.0 (13.0)	68.0 (13.0)	< 0.001	73.0 (13.0)	68.0 (13.0)	< 0.001	73.0 (13.0)	69.0 (13.0)	< 0.001
Body-mass index (kg/m^2^)	22.7 (5.2)	21.8 (4.7)	< 0.001	22.7 (4.9)	21.7 (4.7)	< 0.001	22.6 (6.3)	22.2 (5.5)	0.079
Smoking status									
Non-smoker	233 (35.1%)	991 (30.6%)	< 0.001	108 (21.6%)	462 (18.0%)	< 0.001	125 (76.2%)	529 (78.0%)	0.803
Ex-smoker	296 (44.6%)	1360 (41.9%)		277 (55.4%)	1281 (50.0%)		19 (11.6%)	79 (11.7%)	
Current smoker	135 (20.3%)	891 (27.5%)		115 (23.0%)	821 (32.0%)		20 (12.2%)	70 (10.3%)	
Hospital admissions in the previous year									
< 2	492 (74.1%)	2460 (75.9%)	0.346	358 (71.6%)	1907 (74.4%)	0.201	134 (81.7%)	553 (81.6%)	1.000
≥ 2	172 (25.9%)	782 (24.1%)		142 (28.4%)	657 (25.6%)		30 (18.3%)	125 (18.4%)	
Emergency visits in the previous year									
< 2	529 (79.7%)	2508 (77.4%)	0.201	388 (77.6%)	1953 (76.2%)	0.527	141 (86.0%)	555 (81.9%)	0.250
≥ 2	135 (20.3%)	734 (22.6%)		112 (22.4%)	611 (23.8%)		23 (14.0%)	123 (18.1%)	
Symptoms									
Increased cough	421 (63.4%)	1916 (59.1%)	0.041	318 (63.6%)	1500 (58.5%)	0.037	103 (62.8%)	416 (61.4%)	0.789
Increased sputum volume	271 (40.8%)	1297 (40.0%)	0.728	209 (41.8%)	1023 (39.9%)	0.455	62 (37.8%)	274 (40.4%)	0.594
Wheezing	574 (86.4%)	2711 (83.6%)	0.071	427 (85.4%)	2133 (83.2%)	0.236	147 (89.6%)	578 (85.3%)	0.167
mMRC	3.0 (1.0)	3.0 (1.0)	0.020	3.0 (1.0)	3.0 (1.0)	0.071	3.0 (1.0)	3.0 (1.0)	0.107
CAT	20.0 (10.0)	19.0 (9.0)	0.045	20.0 (11.0)	19.0 (9.0)	0.096	20.0 (8.0)	19.0 (9.0)	0.283
Post-bronchodilator FEV_1_% predicted	43.2 (24.7)	41.8 (26.9)	0.368	41.4 (23.9)	40.3 (25.6)	0.202	45.8 (24.1)	49.4 (27.8)	0.233
Post-bronchodilator FEV_1_/FVC (%)	52.0 (16.0)	50.0 (17.0)	0.002	50.0 (16.0)	48.0 (16.0)	0.023	56.0 (16.0)	55.0 (16.0)	0.147
Comorbidity									
Respiratory disease									
Asthma	56 (8.4%)	299 (9.2%)	0.554	40 (8.0%)	216 (8.4%)	0.792	16 (9.8%)	83 (12.2%)	0.420
Bronchiectasis	80 (12.0%)	403 (12.4%)	0.796	56 (11.2%)	296 (11.5%)	0.878	24 (14.6%)	107 (15.8%)	0.722
Lung cancer	4 (0.6%)	48 (1.5%)	0.092	3 (0.6%)	42 (1.6%)	0.101	1 (0.6%)	6 (0.9%)	1.000
Pulmonary artery hypertension	62 (9.3%)	280 (8.6%)	0.598	43 (8.6%)	232 (9.0%)	0.798	19 (11.6%)	48 (7.1%)	0.075
SAS	7 (1.1%)	39 (1.2%)	0.846	7 (1.4%)	34 (1.3%)	1.000	0 (0.0%)	5 (0.7%)	0.589
Pneumonia	201 (30.3%)	1004 (31.0%)	0.747	153 (30.6%)	779 (30.4%)	0.958	48 (29.3%)	225 (33.2%)	0.354
Cardiovascular disease									
Hypertension	316 (47.6%)	993 (30.6%)	< 0.001	235 (47.0%)	772 (30.1%)	< 0.001	81 (49.4%)	221 (32.6%)	< 0.001
Heart failure	72 (10.8%)	118 (3.6%)	< 0.001	53 (10.6%)	91 (3.5%)	< 0.001	19 (11.6%)	27 (4.0%)	< 0.001
Arrhythmia	88 (13.3%)	152 (4.7%)	< 0.001	68 (13.6%)	130 (5.1%)	< 0.001	20 (12.2%)	22 (3.2%)	< 0.001
Digestive disease									
Gastroesophageal reflux disease	18 (2.7%)	58 (1.8%)	0.123	14 (2.8%)	48 (1.9%)	0.222	4 (2.4%)	10 (1.5%)	0.492
Peptic ulcer	15 (2.3%)	57 (1.8%)	0.427	10 (2.0%)	52 (2.0%)	1.000	5 (3.0%)	5 (0.7%)	0.029
Chronic gastritis	38 (5.7%)	133 (4.1%)	0.076	29 (5.8%)	105 (4.1%)	0.094	9 (5.5%)	28 (4.1%)	0.523
Cerebrovascular disease	77 (11.6%)	194 (6.0%)	< 0.001	64 (12.8%)	154 (6.0%)	< 0.001	13 (7.9%)	40 (5.9%)	0.369
Endocrine and metabolic disease									
Diabetes	110 (16.6%)	296 (9.1%)	< 0.001	86 (17.2%)	237 (9.2%)	< 0.001	24 (14.6%)	59 (8.7%)	0.028
Osteoporosis	6 (0.9%)	36 (1.1%)	0.690	6 (1.2%)	24 (0.9%)	0.617	0 (0.0%)	12 (1.8%)	0.137
Other malignant tumors	12 (1.8%)	54 (1.7%)	0.869	10 (2.0%)	47 (1.8%)	0.856	2 (1.2%)	7 (1.0%)	0.690
Pre-admission non-drug therapy									
Pulmonary rehabilitation	70 (10.5%)	312 (9.6%)	0.473	53 (10.6%)	247 (9.6%)	0.511	17 (10.4%)	65 (9.6%)	0.769
Home oxygen therapy	82 (12.3%)	457 (14.1%)	0.241	71 (14.2%)	374 (14.6%)	0.836	11 (6.7%)	83 (12.2%)	0.052
Noninvasive ventilation	10 (1.5%)	38 (1.2%)	0.561	8 (1.6%)	31 (1.2%)	0.511	2 (1.2%)	7 (1.0%)	0.690
Pre-admission medication									
LAMA	227 (34.2%)	1103 (34.0%)	0.964	197 (39.4%)	914 (35.6%)	0.115	30 (18.3%)	189 (27.9%)	0.013
LABA	211 (31.8%)	1035 (31.9%)	0.964	183 (36.6%)	850 (33.2%)	0.148	28 (17.1%)	185 (27.3%)	0.009
ICS	229 (34.5%)	1090 (33.6%)	0.685	191 (38.2%)	895 (34.9%)	0.168	38 (23.2%)	195 (28.8%)	0.173
OCS	9 (1.4%)	102 (3.1%)	0.014	9 (1.8%)	84 (3.3%)	0.087	0 (0.0%)	18 (2.7%)	0.032

Data are presented as n (%) or median (IQR)

*CAD* coronary artery disease, *mMRC* modified Medical Research Council, *CAT* COPD Assessment Test, *FEV*_*1*_ forced expiratory volume in 1 s, *FVC* forced vital capacity, *SAS* sleep apnea syndrome, *LAMA* long-acting muscarinic receptor antagonist, *LABA* long-acting beta-adrenoceptor agonist, *ICS* inhaled corticosteroids, *OCS* oral corticosteroids

The prevalence of CAD was 16.3% in men and 19.5% in women (*P* = 0.034) (Table 1). The age and prevalence of cardiovascular and diabetic comorbidities were higher in patients with CAD for both men and women. However, in men, BMI and the proportion of ex-smokers and patients with increased cough or cerebrovascular comorbidity were significantly higher in subjects with CAD than in those without CAD, which was not the case in women. Compared with men, women used less long-acting muscarinic receptor antagonist (LAMA), long-acting beta-adrenoceptor agonist (LABA), and inhaled corticosteroids (ICS) before admission (all *P* < 0.001). Further analyses showed that only in women, comorbid CAD was associated with less use of pre-admission LAMA and LABA (Table 1).

### Examinations and treatments during hospitalization

As for laboratory tests during hospitalization, patients with CAD had higher values of eosinophils, blood urea nitrogen, creatinine, blood glucose, and NT-proBNP. During hospitalization, short-acting bronchodilator (SABD), LABA, ICS, and systemic corticosteroids were all prescribed more often to patients without CAD than to those with CAD. Patients with comorbid CAD had a higher rate of ICU admission (*P* = 0.022). Details are shown in Table 2.

**Table 2:** Examinations and treatments during hospitalization of the study patients according to status of coronary artery disease and sex

Variables, N (missing)^a^	CAD (n = 664)	No CAD (n = 3242)	*P*-value	Men			Women		
				CAD (n = 500)	No CAD (n = 2564)	*P*-value	CAD (n = 164)	No CAD (n = 678)	*P*-value
Heart rate (beats per min), 4	84.0 (20.0)	87.0 (19.0)	< 0.001	84.0 (22.0)	87.0 (20.0)	< 0.001	85.0 (18.0)	87.0 (18.0)	0.131
Respiratory rate (breaths per min), 12	20.0 (2.0)	20.0 (2.0)	0.595	20.0 (2.0)	20.0 (2.0)	0.705	20.0 (2.0)	20.0 (2.0)	0.634
Blood gas^b^, 1983									
pH	7.42 (0.05)	7.41 (0.05)	0.430	7.41 (0.05)	7.41 (0.05)	0.941	7.42 (0.04)	7.42 (0.05)	0.326
PaO_2_ (mmHg)	71.0 (19.0)	70.0 (19.0)	0.428	70.9 (19.0)	71.0 (19.0)	0.511	71.0 (19.0)	68.0 (18.0)	0.445
PaCO_2_ (mmHg)	41.2 (9.0)	41.4 (10.0)	0.426	41.0 (9.0)	41.4 (10.0)	0.290	42.0 (10.0)	41.8 (9.0)	0.929
Blood routine, 48									
WBCs (× 10^9^/L)	7.20 (3.83)	7.21 (3.75)	0.451	7.38 (3.82)	7.26 (3.84)	0.447	7.01 (3.90)	7.12 (3.54)	0.739
Neutrophils (%)	70.0 (15.5)	70.1 (17.6)	0.737	71.3 (16.0)	70.3 (17.8)	0.554	68.6 (13.2)	69.5 (16.8)	0.823
Lymphocytes (%)	19.0 (13.0)	18.7 (14.6)	0.698	18.1 (12.7)	18.2 (14.7)	0.500	21.2 (12.5)	20.9 (14.0)	0.983
NLR	3.7 (3.7)	3.7 (4.3)	0.744	3.8 (4.1)	3.9 (4.6)	0.545	3.3 (2.8)	3.3 (3.1)	0.900
Eosinophils (%)	1.6 (2.6)	1.4 (2.8)	0.030	1.6 (2.7)	1.5 (2.9)	0.037	1.4 (2.5)	1.3 (2.5)	0.404
Red blood cells (× 10^12^/L)	4.48 (1.00)	4.52 (1.00)	0.063	4.51 (1.00)	4.58 (1.00)	0.033	4.42 (1.00)	4.35 (1.00)	0.538
Hemoglobins (g/L)	136.0 (22.0)	138.0 (24.0)	0.056	137.0 (22.0)	140.0 (24.0)	0.028	131.0 (19.0)	130.0 (21.0)	0.257
Platelets (× 10^9^/L)	199.0 (80.0)	208.0 (96.0)	0.127	196.0 (80.5)	204.0 (97.0)	0.116	208.0 (92.5)	219.5 (90.8)	0.501
Blood biochemistry, 127									
ALT (U/L)	16.0 (11.8)	16.6 (12.0)	0.160	17.0 (12.4)	17.0 (12.9)	0.298	15.0 (9.2)	15.0 (11.0)	0.653
AST (U/L)	19.0 (9.0)	19.5 (9.5)	0.037	19.2 (9.7)	19.7 (9.4)	0.250	17.5 (8.7)	19.0 (10.0)	0.040
BUN (mmol/L)	5.7 (2.9)	5.4 (2.6)	< 0.001	5.8 (2.9)	5.5 (2.6)	0.001	5.1 (2.6)	4.8 (2.4)	0.029
Cr (μmol/L)	72.0 (29.3)	70.4 (23.1)	0.002	76.5 (29.8)	73.0 (21.3)	< 0.001	59.2 (23.0)	58.7 (19.9)	0.675
Blood glucose (mmol/L), 137	5.6 (1.9)	5.5 (1.8)	0.020	5.7 (2.0)	5.5 (1.9)	0.037	5.6 (1.8)	5.4 (1.7)	0.251
NT-proBNP (pg/ml), 2997	230.6 (584.0)	113.0 (220.0)	< 0.001	236.0 (577.0)	111.0 (219.0)	< 0.001	201.0 (558.0)	119.0 (230.0)	0.187
CRP (mg/L), 1610	5.0 (12.0)	4.7 (15.0)	0.793	5.0 (15.0)	5.0 (16.0)	0.950	4.3 (9.0)	4.3 (11.0)	0.731
PCT (ng/ml), 1693	0.06 (0.10)	0.05 (0.08)	0.610	0.06 (0.10)	0.05 (0.08)	0.727	0.05 (0.07)	0.05 (0.07)	0.660
SABD, 0	472 (71.1%)	2447 (75.5%)	0.019	357 (71.4%)	1941 (75.7%)	0.042	115 (70.1%)	506 (74.6%)	0.277
LAMA, 0	53 (8.0%)	334 (10.3%)	0.074	44 (8.8%)	271 (10.6%)	0.260	9 (5.5%)	63 (9.3%)	0.123
LABA, 0	50 (7.5%)	387 (11.9%)	0.001	41 (8.2%)	309 (12.1%)	0.014	9 (5.5%)	78 (11.5%)	0.031
ICS, 0	71 (10.7%)	441 (13.6%)	0.043	56 (11.2%)	350 (13.7%)	0.149	15 (9.1%)	91 (13.4%)	0.151
Nebulized corticosteroids, 0	438 (66.0%)	2061 (63.6%)	0.249	324 (64.8%)	1613 (62.9%)	0.447	114 (69.5%)	448 (66.1%)	0.408
Systemic corticosteroids, 0	180 (27.1%)	1091 (33.7%)	0.001	144 (28.8%)	876 (34.2%)	0.022	36 (22.0%)	215 (31.7%)	0.017
Antibiotics, 0	577 (86.9%)	2807 (86.6%)	0.851	426 (85.2%)	2208 (86.1%)	0.622	151 (92.1%)	599 (88.3%)	0.209
ICU admission, 0	13 (2.0%)	29 (0.9%)	0.022	12 (2.4%)	25 (1.0%)	0.013	1 (0.6%)	4 (0.6%)	1.000

Data are presented as n (%) or median (IQR). ^a^The number of missing values for each variable. ^b^Blood gas was measured under the condition of breathing in room air

*CAD* coronary artery disease, *PaO*_*2*_ partial pressure of oxygen, *PaCO*_*2*_ partial pressure of carbon dioxide, *WBCs* white blood cells, *NLR* neutrophil-to-lymphocyte ratio, *ALT* alanine aminotransferase, *AST* aspartate aminotransferase, *BUN* blood urea nitrogen, *Cr* creatinine, *NT-proBNP* N-terminal pro-B-type natriuretic peptide, *CRP* C-reactive protein, *PCT* procalcitonin, *SABD* short-acting bronchodilator, *LAMA* long-acting muscarinic receptor antagonist, *LABA* long-acting beta-adrenoceptor agonist, *ICS* inhaled corticosteroids, *ICU* intensive care unit

Only in men, comorbid CAD was significantly related to higher levels of eosinophils, creatinine, blood glucose, and NT-proBNP (Table 2). As for treatments during hospitalization, in men, patients with CAD were less likely to be prescribed SABD than those without (*P* = 0.042), but the difference was not statistically significant between the two groups in women (*P* = 0.277). Additionally, the rate of ICU admission was higher in cases with CAD compared to those without in men (*P* = 0.013) but similar between the two groups in women (*P* = 1.000) (Table 2).

### Sex differences in the predictors of comorbid CAD

In men, age, BMI, hypertension, heart failure, arrhythmia, cerebrovascular disease, and diabetes were independently associated with the prevalence of CAD in AECOPD patients (Table 3). However, in women, multivariate analysis indicated that cerebrovascular disease and diabetes were weakly linked to comorbid CAD. Pre-admission use of LABA and home oxygen therapy indicated a lower possibility of comorbid CAD in women (Table 3).

**Table 3:** Multivariate logistic regression analyses of factors associated with comorbid coronary artery disease in men and women

Variables^a^	Coefficient *B*	Standard error	Wald	*P*-value	OR	95% CI lower	95% CI upper
Men							
Age (years)	0.042	0.006	48.436	< 0.001	1.043	1.030	1.055
Body-mass index (kg/m^2^)	0.048	0.015	10.773	0.001	1.049	1.019	1.079
Hypertension	0.430	0.106	16.289	< 0.001	1.537	1.247	1.893
Heart failure	0.889	0.193	21.252	< 0.001	2.433	1.667	3.550
Arrhythmia	0.659	0.170	14.954	< 0.001	1.933	1.384	2.699
Cerebrovascular disease	0.558	0.164	11.508	0.001	1.747	1.266	2.412
Diabetes	0.497	0.145	11.718	0.001	1.643	1.236	2.183
Constant	-6.022	0.562	114.621	< 0.001			
Women							
Age (years)	0.044	0.010	17.562	< 0.001	1.045	1.024	1.066
Pre-admission home oxygen therapy	− 0.840	0.358	5.496	0.019	0.432	0.214	0.871
Pre-admission LABA	− 0.621	0.242	6.593	0.010	0.537	0.335	0.863
Hypertension	0.668	0.187	12.759	< 0.001	1.951	1.352	2.814
Heart failure	1.024	0.344	8.837	0.003	2.784	1.417	5.468
Arrhythmia	1.207	0.339	12.688	< 0.001	3.343	1.721	6.496
Constant	− 4.731	0.747	40.084	< 0.001			

*OR* odds ratio, *CI* confidence interval, *LABA* long-acting beta-adrenoceptor agonist

^a^Only significant variables of baseline characteristics are listed

### Clinical outcomes during hospitalization

**Fig. 2:**
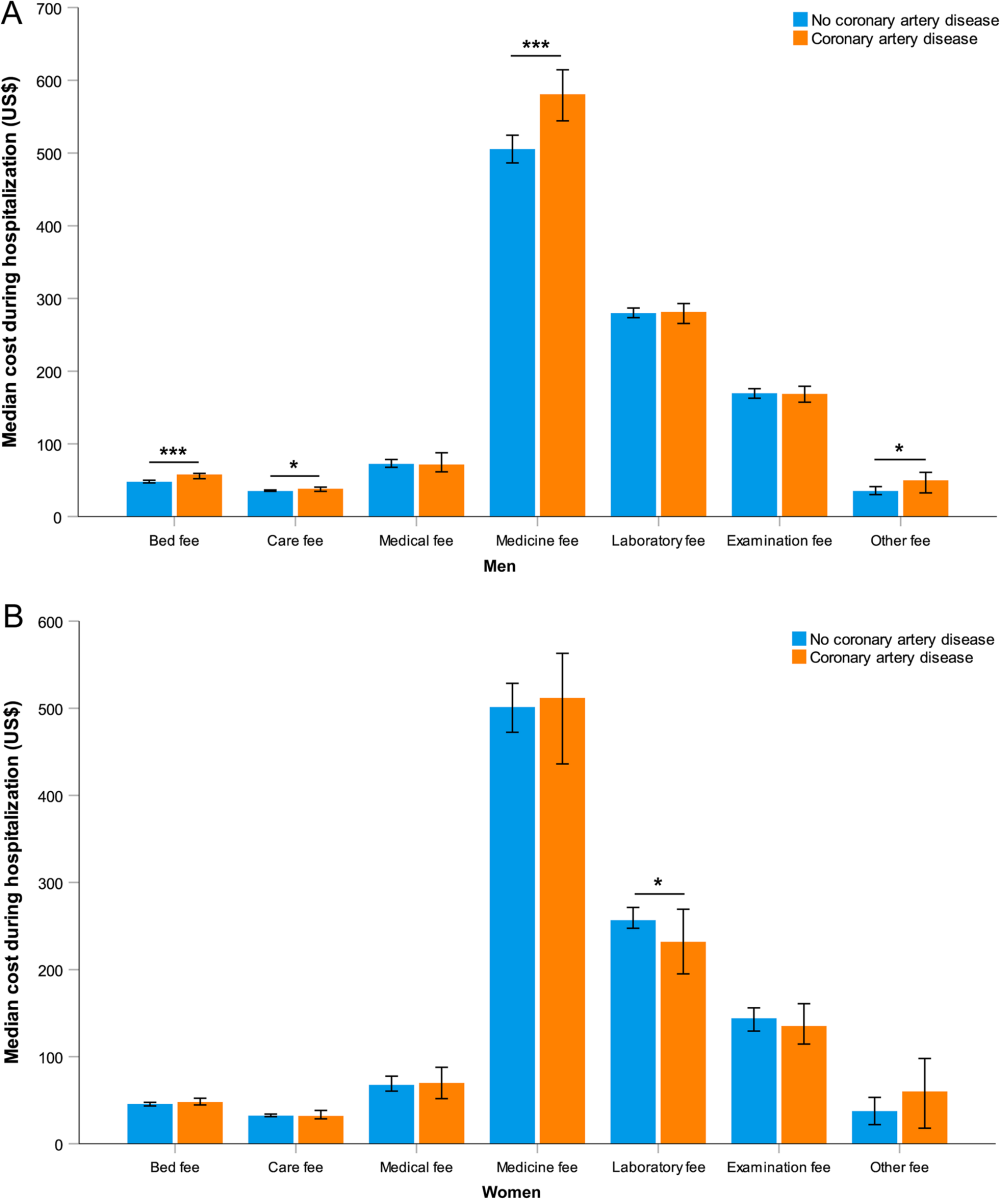
Various cost during hospitalization in men (**A**) and women (**B**) according to status of coronary artery disease. Statistically significant differences between groups are indicated as *P ≤ 0.05, **P ≤ 0.01, and ***P ≤ 0.001. Error bars show 95% confidence interval

For all patients enrolled in our study, comorbid CAD was associated with a longer length of hospital stay and higher total cost (Table 4). In men, the median time in hospital was significantly longer in those with CAD than in those without CAD (median 10.0 days vs. 9.0 days,* P* < 0.001), whereas no statistical difference was found between the two groups in women (Table 4). Similarly, the total cost during hospitalization was significantly higher only in men with CAD than in those without CAD (median $1502.2 vs. $1373.4,* P* < 0.001) (Table 4). In particular, medicine fee in men was numerically higher for those with CAD (Fig. 2A). However, in women, almost all types of costs during hospitalization showed no significant differences between the two groups (Fig. 2B). As for the change of CAT score at discharge compared to admission, only men showed a significant difference between those with and without CAD (Table 4).

**Table 4:** Clinical outcomes of the study patients according to status of coronary artery disease and sex

Clinical outcomes	CAD (n = 664)	No CAD (n = 3242)	*P*-value	Men			Women		
				CAD (n = 500)	No CAD (n = 2564)	*P*-value	CAD (n = 164)	No CAD (n = 678)	*P*-value
Length of hospital stay (days)	10.0 (5.8)	9.0 (4.0)	< 0.001	10.0 (5.0)	9.0 (5.0)	< 0.001	9.0 (5.0)	9.0 (4.0)	0.122
Total cost during hospitalization (US$)	1419.3 (952.4)	1352.1 (862.1)	0.002	1502.2 (987.3)	1373.4 (886.6)	< 0.001	1197.0 (812.3)	1289.4 (756.6)	0.421
Change of CAT at discharge compared to admission	− 8.0 (9.0)	− 7.0 (9.0)	0.003	− 8.0 (9.0)	− 7.0 (9.0)	0.006	− 8.0 (8.0)	− 7.0 (9.0)	0.235
CAT at day 30	11.0 (9.0)	11.0 (8.0)	0.020	12.0 (9.0)	11.0 (8.0)	0.046	11.0 (7.0)	10.0 (9.0)	0.194
mMRC at day 30	2.0 (1.0)	1.0 (1.0)	< 0.001	2.0 (1.0)	1.0 (1.0)	0.003	2.0 (1.0)	1.0 (1.0)	0.007
St George’s Respiratory Questionnaire at day 30									
Symptoms	52.4 (21.0)	53.6 (18.0)	0.225	52.6 (20.0)	54.0 (18.0)	0.659	49.3 (19.0)	52.2 (18.0)	0.108
Activities	41.8 (31.0)	42.6 (30.0)	0.812	41.8 (37.0)	47.2 (30.0)	0.868	42.3 (37.0)	41.8 (30.0)	0.870
Impacts	14.2 (25.0)	14.1 (24.0)	0.936	14.3 (25.0)	14.1 (24.0)	0.832	12.9 (23.0)	14.3 (23.0)	0.842
Total	29.3 (23.0)	30.0 (22.0)	0.764	29.7 (23.0)	30.3 (22.0)	0.986	27.9 (21.0)	29.6 (22.0)	0.595
All-cause readmission or death within 30 days	13 (3.1%)	68 (3.4%)	0.770	11 (3.4%)	59 (3.7%)	0.872	2 (2.0%)	9 (2.2%)	1.000
AECOPD readmission within 30 days	11 (2.7%)	47 (2.4%)	0.861	9 (2.8%)	40 (2.5%)	0.846	2 (2.1%)	7 (1.7%)	0.686

*CAD* coronary artery disease, *CAT* COPD Assessment Test, *mMRC* modified Medical Research Council, *AECOPD* acute exacerbations of chronic obstructive pulmonary disease

Data are presented as n (%) or median (IQR)

### Clinical outcomes within 30 days after discharge

A total of 2407 (61.6%) patients had follow-up data within 30 days after discharge. Baseline characteristics were largely similar among patients with and without follow-up data (see Additional file 1: Table S1). Subjects with comorbid CAD had a higher mMRC dyspnea grade at day 30 than those without CAD in both men and women, but only in men, comorbid CAD was associated with a higher CAT score at day 30 (Table 4). No significant difference was found for the SGRQ score (Table 4).

The 30-day all-cause readmission or death and AECOPD readmission occurred in 3.4% and 2.4% of the 2407 patients, respectively. The all-cause readmission or death rates and the AECOPD readmission rates showed no differences between patients with and without CAD regardless of sex (Table 4). Cox regression analyses showed that sex and CAD were not statistically significant risk factors for all-cause readmission or death (Table 5) or AECOPD readmission (see Additional file 1: Table S2). Further analysis indicated that the number of hospital admissions ≥ 2 in the previous year contributed independently to a higher risk of readmission or death, whereas higher FEV_1_% predicted had a protective effect against 30-day readmission or death.

**Table 5:** Univariate and multivariate Cox regression analyses of factors associated with all-cause readmission or death within 30 days

Variables	Crude HR (95% CI)	*P*-value	Adjusted HR (95% CI)^a^	*P*-value
CAD	0.91 (0.50–1.64)	0.744	–	–
Sex and status of CAD				
Men without CAD	1.00 (reference)	–	–	–
Men with CAD	0.92 (0.48–1.75)	0.793	–	–
Women without CAD	0.59 (0.29–1.19)	0.142	–	–
Women with CAD	0.54 (0.13–2.23)	0.398	–	–
Age (years)	1.01 (0.99–1.03)	0.381	–	–
Men	1.69 (0.90–3.20)	0.105	–	0.337
Body-mass index (kg/m^2^)	0.95 (0.89–1.01)	0.081	–	0.385
Smoker^b^	1.00 (0.62–1.60)	0.999	–	–
Hospital admissions ≥ 2 in the previous year	2.32 (1.49–3.60)	< 0.001	1.94 (1.24–3.04)	0.004
mMRC ≥ 2	1.65 (0.83–3.30)	0.155	–	–
CAT ≥ 10	1.23 (0.54–2.83)	0.623	–	–
Post-bronchodilator FEV_1_% predicted	0.97 (0.96–0.99)	< 0.001	0.98 (0.96–0.99)	0.001
Comorbidity				
Respiratory diseases	1.24 (0.80–1.93)	0.334	–	–
Cardiovascular diseases^c^	1.39 (0.90–2.14)	0.142	–	–
Digestive diseases	1.35 (0.65–2.81)	0.416	–	–
Cerebrovascular disease	1.53 (0.77–3.07)	0.226	–	–
Endocrine and metabolic diseases	1.30 (0.70–2.39)	0.406	–	–
Other malignant tumors	1.50 (0.37–6.10)	0.572	–	–
Laboratory data during hospitalization				
PaO_2_ (mmHg)	1.00 (0.99–1.01)	0.540	–	–
PaCO_2_ (mmHg)	1.02 (1.00–1.03)	0.110	–	–
NLR	1.01 (1.00–1.01)	< 0.001	1.01 (1.00–1.01)	< 0.001
Eosinophils (%)	0.95 (0.87–1.03)	0.210	–	–
NT-proBNP (pg/ml)	1.00 (1.00–1.00)	0.936	–	–
CRP (mg/L)	0.99 (0.99–1.00)	0.270	–	–
PCT (ng/ml)	0.84 (0.47–1.51)	0.563	–	–
Treatment during hospitalization				
SABD	0.89 (0.54–1.46)	0.641	–	–
LAMA	0.90 (0.41–1.94)	0.780	–	–
LABA	0.71 (0.33–1.53)	0.378	–	–
ICS	0.61 (0.28–1.32)	0.212	–	–
Nebulized corticosteroids	1.20 (0.76–1.92)	0.434	–	–
Systemic corticosteroids	2.33 (1.50–3.60)	< 0.001	2.01 (1.30–3.13)	0.002
Antibiotics	1.61 (0.74–3.48)	0.232	–	–

*HR* hazard ratio, *CI* confidence interval, *CAD* coronary artery disease, *mMRC* modified Medical Research Council, *CAT* COPD Assessment Test, *FEV*_*1*_ forced expiratory volume in 1 s, *PaO*_*2*_ partial pressure of oxygen, *PaCO*_*2*_ partial pressure of carbon dioxide, *NLR* neutrophil-to-lymphocyte ratio, *NT-proBNP* N-terminal pro-B-type natriuretic peptide, *CRP* C-reactive protein, *PCT* procalcitonin, *SABD* short-acting bronchodilator, *LAMA* long-acting muscarinic receptor antagonist, *LABA* long-acting beta-adrenoceptor agonist, *ICS* inhaled corticosteroids

^a^The multivariable model was adjusted for sex, BMI, hospital admissions in the previous year, post-bronchodilator FEV_1_% predicted, NLR, and systemic corticosteroids during hospitalization

^b^Smoker refers to the subject who has a history of smoking

^c^CAD as a separate variable is not included in the cardiovascular diseases

## Discussion

To the best of our knowledge, this is the first and largest study using real-world data to investigate sex differences in AECOPD outcomes according to the presence or absence of CAD. We found that 17.0% of patients admitted with AECOPD had concomitant CAD and this frequency was higher in women in our study. The predictors of comorbid CAD showed obvious sex difference. In addition, only in men, comorbid CAD was associated with a longer length of hospital stay and higher total cost. For women, comorbid CAD did not significantly influence the clinical outcomes. Sex and CAD were not relevant to readmissions or death within 30 days after discharge.

COPD characterized by low-grade systemic inflammation plays a role in the development or acceleration of CVD [17]. A large meta-analysis reported that COPD patients had a two times higher risk of CAD [18]. However, CAD prevalence rates in COPD patients range from 3 to 64% [18], partly due to the different severity of COPD and also because pulmonary symptoms can mimic and mask the symptoms of CVD leading to misdiagnosis or missed diagnosis. Although studies have showed that exacerbations confer an increased risk of subsequent cardiovascular events [19], data about the prevalence of CAD in AECOPD are limited. The prevalence rate in this study was similar to 17.0% reported by Almagro et al. [11], but lower than 28.8% reported by Aliyali et al. who recruited older participants aged over 50 in their study [7]. Contrary to the results found by Almagro et al. [11], the prevalence of CAD in our study was higher in women than in men. This might be related to insufficient pre-admission treatment in women including the use of LABA and home oxygen therapy acting as protective factors against comorbid CAD. In fact, a study in hospitalized COPD patients in China has reported that for CAD, the prevalence in men decreased and that for women increased year by year, which could be partly explained by the increase of occupational exposure and social or psychological stress in women [12].

Common cardiovascular risk factors including age, BMI, hypertension, and diabetes were also independently associated with the prevalence of CAD in men admitted for AECOPD in our study. The results were in line with the findings of Bellocchia et al. that age and BMI were predictive factors of CAD, although their study recruited patients with stable COPD [20]. In addition, both hypertension and diabetes can cause structural alterations of lung and heart tissue due to systemic inflammation or oxidative stress, which were highly associated not only with the development of CVD but also with the pathogenesis of COPD [21]. Moreover, cerebrovascular disease and other types of CVD might be independently associated with comorbid CAD considering a number of shared risk factors (e.g., age and obesity) between them.

In women, the role of BMI, cerebrovascular disease, and diabetes became less important when assessing the risk of comorbid CAD. Similarly, a large study based on 2046 stable COPD patients reported that in men, age, BMI, smoking status, mMRC, energy, and pulmonary function were related to cardiac disease, while in women the predictors only included age [8]. Of note, we found pre-admission use of LABA and home oxygen therapy were protective factors against comorbid CAD in women but showed no relationship with comorbid CAD in men. Although evidence for the safety of LABA in patients with concomitant COPD and CVD is less definitive, many studies including randomized controlled trials and post-hoc analyses have concluded that LABA administration does not increase the risk of cardiovascular events in patients with COPD [22, 23]. There are also data suggesting that LABA can produce a positive impact on reducing the risk of CVD. An interventional, randomized, double-blind clinical trial showed that a clinically relevant improvement of dyspnea with indacaterol was associated with a significant increase of the right ventricular compliance indexes [24]. Inhaled LABA also had direct benefits on pulmonary haemodynamics [25] and reduced exacerbations in COPD patients [26], which could lead to an increased risk of CVD [19]. The reason for different roles of LABA in men and women was not clear but might be partly explained by the sex differences in response to LABA.

In our study, comorbid CAD was associated with poorer short-term outcomes. This was consistent with previous studies. Aliyali et al. reported that the median length of stay was 7 days in patients with CAD versus 6 days for patients without CAD and the adjusted odds ratio for the risk of ICU admission in patients with CAD was 2.97 [7]. Another study analyzing stable-state data of 386 subjects from the London COPD Cohort showed that patients with CAD had significantly worse health status, lower exercise capacity, and more dyspnea as well as longer exacerbations [5]. However, as far as we know, no research has investigated the sex-related differences in the effect of comorbid CAD on AECOPD outcomes. We found that only in men, comorbid CAD was associated with a higher rate of ICU admission and longer length of hospital stay. The following reasons may account for the poorer outcomes in men with CAD. For the population in the ACURE study, only in men, patients with CAD had a higher level of NT-proBNP proposed as a marker of left ventricular and endothelial dysfunction and early mortality in patients with AECOPD [27]. The proportion of patients prescribed SABD during hospitalization was significantly lower in men with CAD than those without. Less use of SABDs, which were recommended as the initial bronchodilators for AECOPD [1], could result in inadequate symptom control.

Several studies demonstrated a substantial economic burden associated with comorbid CVD among COPD patients. In a nationally representative population of COPD adults in the United States, presence of CVD was associated with higher annual healthcare expenditure and resource utilization [28]. A population study using health administrative data for over 7 million people in Canada reported that individuals with COPD had more health service claims including higher claim rates of emergency department visits and hospitalizations for CVD compared to the general population [29].

The impact of comorbid CAD on the economic burden during an AECOPD and whether it differs by sex remain unclear. Our study showed that patients with CAD had a higher economic burden of hospitalization than those without CAD, but the difference was significant only in men. In particular, accounting for the highest proportion of total cost, medicine fee was also numerically higher in men with CAD. This might be related to their serious conditions indicated by the higher rate of ICU admission and longer length of hospital stay.

In our analyses, COPD symptoms assessed by the CAT score and mMRC dyspnea grade were more severe in patients with CAD at day 30, especially in men with CAD, although the decreases of CAT score during hospitalization were more significant among patients with CAD. No relationship was found between comorbid CAD and all-cause readmission or death or AECOPD readmission within 30 days regardless of sex. To our best knowledge, there is no relevant research comparing the risk of readmission or death after 30 days between AECOPD patients with and without comorbid CAD.

A prospective observational study in 2887 COPD patients enrolled from primary care claimed that there was no difference in the annualized rate of AECOPD and mortality between those with or without CVD during the 24-month follow-up period and sensitivity analyses for each CVD diagnosis also did not show any relationship between individual CVD and the incidence of exacerbations [30]. Patel et al. recruiting patients from the London COPD Cohort conducted a prospective evaluation of exacerbations in those who had completed symptom diaries for ≥ 1 year and reported a longer duration but not an increased frequency of AECOPD in patients with CAD [5]. However, when the follow-up period was extended to more than 5 years, data from two population-based cohorts showed that the presence of respiratory impairment and comorbid CVD predicted higher mortality and higher risk of all-cause hospitalization [31]. In addition, in our real-world study with relatively recent recruitment of AECOPD patients, variables associated with a higher risk of 30-day readmission or death were frequent hospitalizations and lower FEV_1_% predicted.

Indeed, data on differences between the sexes in COPD with comorbidities are scarce. In patients with COPD, it has been suggested that there are substantial differences between men and women in airway anatomy, with women exhibiting smaller lumina and disproportionately thicker airway walls than men [32]. Furthermore, important differences in biology, genetic susceptibility to airway damage, and lung microbiome have been reported [33]. These differences between men and women with COPD may have a role in differently influencing the characteristics of COPD comorbidities. In fact, the protective role of estrogens in regulating the contractility of airway smooth muscles [34] and in the cardiovascular system [35] probably reduces the adverse effects of comorbid CAD on women. The potential differences in prognosis and complicated mechanisms need to be further studied.

The strength of our study was that we used real-world data from the ACURE study which was the first and largest registry of hospitalized AECOPD patients in China. Our analyses provided a comprehensive overview of the clinical characteristics and outcomes of in-hospital AECOPD patients. Moreover, all patients had a diagnosis of COPD confirmed by spirometry at baseline. This study also had several limitations. First, data on treatments for CAD before and during hospitalization were unavailable in the ACURE study, which could also affect clinical outcomes. Second, patients in our study might be admitted to the hospital at different periods of their exacerbations and there was no accurate data regarding when their symptoms started. Some patients might be admitted earlier in their exacerbation course, which potentially confounded the results. However, this real-world study reflected the realities of patients requiring hospitalization and provided valuable information for clinical practice. Such data collection will be planned in our future studies. Third, there were missing data (e.g., blood gas) in a portion of patients. Fourth, not all patients in our study had follow-up data within 30 days after discharge. However, the baseline characteristics were largely similar between those with or without follow-up data, making selective bias less likely. Finally, no long-term prognostic analysis was performed because the ACURE study is still ongoing with only the 30-day data available.

## Conclusions

Using data from a large AECOPD cohort, we revealed the different role of clinical characteristics in men and women in evaluating the risk of comorbid CAD. For men hospitalized for AECOPD, comorbid CAD was associated with a higher rate of ICU admission, longer length of hospital stay, higher economic burden of hospitalization, and more severe COPD symptoms at day 30. No significant difference was found in women. Comorbid CAD showed no relationship with 30-day readmission or death regardless of sex. Clinicians should have heightened attention for men with comorbid CAD to achieve an optimal management of AECOPD patients.

## Supplementary Information

**Additional file 1: Table S1.** Baseline characteristics of the patients with or without follow-up data. **Table S2.** Univariate and multivariate Cox regression analyses of factors associated with AECOPD readmission within 30 days.

## Data Availability

The datasets used and/or analyzed during the current study are available from the corresponding author on reasonable request.

## Abbreviations

CAD: Coronary artery disease
COPD: Chronic obstructive pulmonary disease
AECOPD: Acute exacerbation of COPD
ACURE: Acute exacerbation of chronic obstructive pulmonary disease inpatient registry
ICU: Intensive care unit
CVD: Cardiovascular disease
FEV1: Forced expiratory volume in 1 s
FVC: Forced vital capacity
BMI: Body-mass index
mMRC: Modified Medical Research Council
CAT: COPD assessment test
SGRQ: St George’s respiratory questionnaire
IQR: Interquartile range
SD: Standard deviation
NLR: Neutrophil-to-lymphocyte ratio
NT-proBNP: N-terminal pro-B-type natriuretic peptide
CRP: C-reactive protein
PCT: Procalcitonin
LAMA: Long-acting muscarinic receptor antagonist
LABA: Long-acting beta-adrenoceptor agonist
ICS: Inhaled corticosteroids
SAS: Sleep apnea syndrome
OCS: Oral corticosteroids
SABD: Short-acting bronchodilator
PaO2: Partial pressure of oxygen
PaCO2: Partial pressure of carbon dioxide
WBCs: White blood cells
ALT: Alanine aminotransferase
AST: Aspartate aminotransferase
BUN: Blood urea nitrogen
Cr: Creatinine
OR: Odds ratio
CI: Confidence interval
HR: Hazard ratio
