# Supplementary material for: Clinical and economic burden of comorbid coronary artery disease in patients with acute exacerbation of chronic obstructive pulmonary disease: sex differences in a nationwide cohort study
Source: Respir Res. 2022 Feb 12;23:28. doi: 10.1186/s12931-022-01945-7 (PMC8840293; doi:10.1186/s12931-022-01945-7)
Supplement: Supplementary file 1 — Additional file 1: Table S1. Baseline characteristics of the patients with or without follow-up data. Table S2. Univariate and multivariate Cox regression analyses of factors associated with AECOPD readmission within 30 days. [file 12931_2022_1945_MOESM1_ESM.docx]

**Table S1 Baseline characteristics of the patients with or without follow-up data**

| **Variables** | **With follow-up data**  **(n=2407)** | **Without follow-up data**  **(n=1499)** | ***P*-value** |
| --- | --- | --- | --- |
| Age (years) | 70.0 (13.0) | 69.0 (13.0) | 0.061 |
| Men | 1905 (79.1%) | 1159 (77.3%) | 0.187 |
| Body-mass index (kg/m^2^) | 22.0 (4.8) | 22.0 (5.0) | 0.350 |
| Smoker^a^ | 1666 (69.2%) | 1016 (67.8%) | 0.357 |
| Hospital admissions in the previous year |  |  | 0.515 |
| < 2 | 1828 (75.9%) | 1124 (75.0%) |  |
| ≥ 2 | 579 (24.1%) | 375 (25.0%) |  |
| Emergency visits in the previous year |  |  | 0.048 |
| < 2 | 1846 (76.7%) | 1191 (79.5%) |  |
| ≥ 2 | 561 (23.3%) | 308 (20.5%) |  |
| mMRC | 3.0 (1.0) | 3.0 (1.0) | 0.001^b^ |
| CAT |  |  | 0.561 |
| < 10 | 216 (9.0%) | 126 (8.4%) |  |
| ≥ 10 | 2191 (91.0%) | 1373 (91.6%) |  |
| Post-bronchodilator FEV_1_% predicted | 42.1 (26.3) | 41.9 (26.4) | 0.613 |
| Post-bronchodilator FEV_1_/FVC (%) | 50.0 (17.0) | 50.0 (18.0) | 0.696 |
| Comorbidity |  |  |  |
| Respiratory diseases | 1240 (51.5%) | 729 (48.6%) | 0.081 |
| Cardiovascular diseases | 1139 (47.3%) | 670 (44.7%) | 0.113 |
| Digestive diseases | 181 (7.5%) | 112 (7.5%) | 1.000 |
| Cerebrovascular disease | 183 (7.6%) | 88 (5.9%) | 0.038 |
| Endocrine and metabolic diseases | 286 (11.9%) | 157 (10.5%) | 0.178 |
| Other malignant tumors | 40 (1.7%) | 26 (1.7%) | 0.899 |

Data are presented as n (%) or median (IQR). ^a^Smoker refers to the subject who has a history of smoking. ^b^Although there were significant differences in the distribution of mMRC, no difference in the median was found between the two groups.

mMRC, modified Medical Research Council; CAT, COPD Assessment Test; FEV_1_, forced expiratory volume in 1 s; FVC, forced vital capacity.

**Table S2 Univariate and multivariate Cox regression analyses of factors associated with AECOPD readmission within 30 days**

| **Variables** | **Crude HR (95% CI)** | ***P*-value** | **Adjusted HR (95% CI)^a^** | ***P*-value** | **Adjusted HR (95% CI)^b^** | ***P*-value** | **Adjusted HR (95% CI)^c^** | ***P*-value** |
| --- | --- | --- | --- | --- | --- | --- | --- | --- |
| Men | 1.44 (0.71-2.94) | 0.311 | – | 0.723 | – | 0.872 | – | – |
| CAD | 1.12 (0.58-2.15) | 0.745 | – | – | – | – | – | – |
| Sex and status of CAD |  |  |  |  |  |  |  |  |
| Men without CAD | 1.00 (reference) | – | – | – | – | – | – | – |
| Men with CAD | 1.11 (0.54-2.28) | 0.780 | – | – | – | – | – | – |
| Women without CAD | 0.68 (0.30-1.51) | 0.344 | – | – | – | – | – | – |
| Women with CAD | 0.82 (0.20-3.39) | 0.783 | – | – | – | – | – | – |
| Age (years) | 1.02 (0.99-1.05) | 0.133 | – | – | – | – | – | – |
| Body-mass index (kg/m^2^) | 0.92 (0.86-1.00) | 0.041 | – | 0.250 | – | – | – | – |
| Smoker^d^ | 0.85 (0.50-1.46) | 0.559 | – | – | – | – | – | – |
| Hospital admissions ≥2 in the previous year | 3.22 (1.92-5.38) | <0.001 | 2.78 (1.65-4.67) | <0.001 | 2.72 (1.56-4.77) | <0.001 | 2.72 (1.56-4.77) | <0.001 |
| mMRC ≥2 | 2.19 (0.88-5.48) | 0.094 | – | 0.326 | – | – | – | – |
| CAT ≥10 | 1.33 (0.48-3.69) | 0.577 | – | – | – | – | – | – |
| Post-bronchodilator FEV_1_% predicted | 0.97 (0.95-0.98) | <0.001 | 0.97 (0.95-0.99) | 0.001 | 0.97 (0.96-0.99) | 0.004 | 0.97 (0.96-0.99) | 0.004 |
| Comorbidity |  |  |  |  |  |  |  |  |
| Respiratory diseases | 1.34 (0.79-2.26) | 0.273 | – | – | – | – | – | – |
| Cardiovascular diseases^e^ | 1.38 (0.83-2.31) | 0.218 | – | – | – | – | – | – |
| Digestive diseases | 1.17 (0.47-2.93) | 0.735 | – | – | – | – | – | – |
| Cerebrovascular disease | 1.96 (0.93-4.14) | 0.076 | 2.21 (1.04-4.66) | 0.038 | 2.48 (1.16-5.31) | 0.019 | 2.48 (1.16-5.31) | 0.019 |
| Endocrine and metabolic diseases | 1.38 (0.68-2.82) | 0.369 | – | – | – | – | – | – |
| Laboratory data during hospitalization |  |  |  |  |  |  |  |  |
| PaO_2_ (mmHg) | 1.00 (0.99-1.01) | 0.996 | – | – | – | – | – | – |
| PaCO_2_ (mmHg) | 1.02 (1.00-1.04) | 0.025 | – | – | – | 0.530 | – | 0.530 |
| NLR | 1.01 (1.00-1.01) | 0.080 | – | – | – | 0.167 | – | 0.167 |
| Eosinophils (%) | 0.92 (0.82-1.03) | 0.143 | – | – | – | – | – | – |
| NT-proBNP (pg/ml) | 1.00 (1.00-1.00) | 0.989 | – | – | – | – | – | – |
| CRP (mg/L) | 1.00 (0.99-1.00) | 0.378 | – | – | – | – | – | – |
| PCT (ng/ml) | 0.91 (0.55-1.50) | 0.702 | – | – | – | – | – | – |
| Treatment during hospitalization |  |  |  |  |  |  |  |  |
| SABD | 0.89 (0.50-1.61) | 0.705 | – | – | – | – | – | – |
| LAMA | 0.71 (0.26-1.95) | 0.501 | – | – | – | – | – | – |
| LABA | 1.03 (0.47-2.27) | 0.944 | – | – | – | – | – | – |
| ICS | 0.89 (0.40-1.96) | 0.776 | – | – | – | – | – | – |
| Nebulized corticosteroids | 1.17 (0.68-2.02) | 0.577 | – | – | – | – | – | – |
| Systemic corticosteroids | 2.15 (1.28-3.60) | 0.004 | – | – | – | – | – | 0.053 |
| Antibiotics | 2.06 (0.75-5.68) | 0.164 | – | – | – | – | – | – |

^a^The multivariable model was adjusted for sex, BMI, hospital admissions in the previous year, mMRC, post-bronchodilator FEV_1_% predicted, and cerebrovascular disease.

^b^The multivariable model was adjusted for sex, hospital admissions in the previous year, post-bronchodilator FEV_1_% predicted, cerebrovascular disease, PaCO_2_, and NLR.

^c^The multivariable model was adjusted for hospital admissions in the previous year, post-bronchodilator FEV_1_% predicted, cerebrovascular disease, PaCO_2_, NLR, and systemic corticosteroids during hospitalization.

^d^Smoker refers to the subject who has a history of smoking. ^e^CAD as a separate variable is not included in the cardiovascular diseases.

AECOPD, acute exacerbations of chronic obstructive pulmonary disease; HR, hazard ratio; CI, confidence interval; CAD, coronary artery disease; mMRC, modified Medical Research Council; CAT, COPD Assessment Test; FEV_1_, forced expiratory volume in 1 s; PaO_2_, partial pressure of oxygen; PaCO_2_, partial pressure of carbon dioxide; NLR, neutrophil-to-lymphocyte ratio; NT-proBNP, N-terminal pro-B-type natriuretic peptide; CRP, C-reactive protein; PCT, procalcitonin; SABD, short-acting bronchodilator; LAMA, long-acting muscarinic receptor antagonist; LABA, long-acting beta-adrenoceptor agonist; ICS, inhaled corticosteroids.
